# Reply: Expression of the proteolysis-inducing factor core-peptide mRNA is upregulated in both tumour and adjacent normal tissue in gastrooesophageal malignancy

**DOI:** 10.1038/sj.bjc.6604117

**Published:** 2007-12-18

**Authors:** D A C Deans, S J Wigmore, H Gilmour, M J Tisdale, K C H Fearon, J A Ross

**Affiliations:** 1 Tissue Injury and Repair Group, Clinical and Surgical Sciences, University of Edinburgh, The Chancellor's Building, 49 Little France Crescent, Edinburgh EH16 4SB, UK

**Sir**,

Extrapolating from our findings that mRNA for the dermcidin gene (Schittek *et al*, 2001), which encodes the proteolysis-inducing factor (PIF) core peptide (PIF-CP), is present in both gastrooesophageal tumour and adjacent non-tumour tissue, EA Rapoport suggests that PIF may be generally involved in muscle breakdown via production of PIF in tissues requiring amino acids for tissue regeneration, for example, in liver regeneration. There are, however, several caveats, which need to be applied to this suggestion. The first is that it is only the glycosylated form of the PIF-CP that has been shown to induce muscle breakdown (Todorov *et al*, 1996), and this form is difficult to identify in tissues (Wieland *et al*, 2007). Glycosylated PIF has also been shown to have pro-inflammatory effects on hepatocytes (Watchorn *et al*, 2001) and other cell types. In our study (Deans *et al*, 2006), we made no claim that glycosylated PIF was present in gastrooesophageal tumours or adjacent tissues. In addition, the PIF-CP is homologous to a neural survival peptide (YP-30) (Cunningham *et al*, 2002), and expression of the gene encoding the PIF-CP has been shown to provide a small survival and proliferative advantage to other cell types including tumours (Lowrie *et al*, 2006, and Stewart *et al*, 2007). Furthermore, evidence suggests that the YP-30/PIF-CP peptide may have a role in development, acting as a maternal blood-borne factor, which promotes survival of the developing thalamus (Landgraf *et al*, 2005). In a recent development, there is now evidence that peptide products of the dermcidin gene may participate in the regulation of placental function by means of modulating proteolytic cascades on the trophoblastic surface (Motoyama *et al*, 2007) and having proteolytic activity. Therefore, while EA Rapoport's suggestion may be unlikely in the light of present evidence, there is still much to be discovered about this fascinating gene and its products.
